# Manic episodes triggered by weight loss in a patient with bipolar disorder: the importance of integrated lifestyle interventions in psychiatric care

**DOI:** 10.1192/j.eurpsy.2025.1053

**Published:** 2025-08-26

**Authors:** D. De Francesco, S. Cipolla, P. Catapano, D. Vozza, R. Monda, M. C. Della Corte, R. Murolo, V. De Santis, F. Perris, F. Catapano

**Affiliations:** 1 Department of Psychiatry, University of Campania “Luigi Vanvitelli”, Naples, Italy

## Abstract

**Introduction:**

Worldwide, obesity has become a major public health issue, affecting over 650 million people, and individuals with severe mental disorders (SMD) are disproportionately affected by higher rates of obesity and metabolic syndrome (MetS). These patients often face significant barriers to improving their lifestyle, including the metabolic side effects of psychiatric medications, which are associated with weight gain, insulin resistance, and dyslipidemia. Moreover, psychiatric patients frequently encounter challenges when seeking care, often due to the stigma surrounding mental illness. This can lead to inadequate medical attention for physical health issues, further exacerbating the vicious cycle between physical and mental health. For these reasons, attention to the physical health of patients with SMD has been increasing in the recent years. However, the attempts to improve physical health can be a trigger by the exacerbation of psychiatric symptoms in more vulnerable patients.

**Objectives:**

The report aims to highlight the reciprocal influence between psychiatric symptoms and physical health, emphasizing the importance of lifestyle interventions in patients with severe mental disorders.

**Methods:**

In this case report, we present a 57-years-old patient with Bipolar I Disorder and class 3 obesity (BMI: 47.689) who experienced recurrent manic episodes triggered by past weight loss attempts, including one involving the use of liraglutide, a glucagon-like peptide-1 (GLP-1) receptor agonist.

**Results:**

To prevent a new manic episode, the patient was admitted to our in-patient ward after he started to take orally liraglutide, a novel drug to treat obesity. As we expected, during hospitalization the patient experienced a manic episode and dysphoric mood, tachypsychia, and sleep disturbance emerged. After a treatment with a combination of valproic acid, risperidone, and gabapentin, the manic episode resolved at time of discharge, but despite the administration of liraglutide, no significant change in body weight was observed.

**Conclusions:**

This case emphasizes the need for a tailored, integrated approach to lifestyle interventions in psychiatric care, in alignment with the “One Health” concept, which highlights the interconnection between physical and mental well-being. However, some patients may require the assistance of a mental health professional while attempting to change their lifestyle during their lifestyle improvement and weight loss attempts, as even modern pharmacological interventions for obesity can act as a psychopathological trigger.

**Disclosure of Interest:**

None Declared

